# A Methodology for Cancer Therapeutics by Systems Pharmacology-Based Analysis: A Case Study on Breast Cancer-Related Traditional Chinese Medicines

**DOI:** 10.1371/journal.pone.0169363

**Published:** 2017-01-09

**Authors:** Yan Li, Jinghui Wang, Feng Lin, Yinfeng Yang, Su-Shing Chen

**Affiliations:** 1 Systems Biology Laboratory, Department of Computer Information Science and Engineering, University of Florida, Gainesville, Florida, United States of America; 2 Key Laboratory of Industrial Ecology and Environmental Engineering (MOE), Faculty of Chemical, Environmental and Biological Science and Technology, Dalian University of Technology, Dalian, Liaoning, P R China; Southern Illinois University School of Medicine, UNITED STATES

## Abstract

Breast cancer is the most common carcinoma in women. Comprehensive therapy on breast cancer including surgical operation, chemotherapy, radiotherapy, endocrinotherapy, etc. could help, but still has serious side effect and resistance against anticancer drugs. Complementary and alternative medicine (CAM) may avoid these problems, in which traditional Chinese medicine (TCM) has been highlighted. In this section, to analyze the mechanism through which TCM act on breast cancer, we have built a virtual model consisting of the construction of database, oral bioavailability prediction, drug-likeness evaluation, target prediction, network construction. The 20 commonly employed herbs for the treatment of breast cancer were used as a database to carry out research. As a result, 150 ingredient compounds were screened out as active molecules for the herbs, with 33 target proteins predicted. Our analysis indicates that these herbs 1) takes a ‘Jun-Chen-Zuo-Shi” as rule of prescription, 2) which function mainly through perturbing three pathways involving the epidermal growth factor receptor, estrogen receptor, and inflammatory pathways, to 3) display the breast cancer-related anti-estrogen, anti-inflammatory, regulation of cell metabolism and proliferation activities. To sum it up, by providing a novel *in silico* strategy for investigation of the botanical drugs, this work may be of some help for understanding the action mechanisms of herbal medicines and for discovery of new drugs from plants.

## Introduction

Over about one million people would have been diagnosed with cancer and 600,000 will have died of cancer during 2004 in the United States [1]. Despite of the substantial improvements in the current treatments that are available for patients diagnosed with cancer and the positive influence of these treatments on survival, the present chemotherapy or radiation therapy still cause an array of traumatic side effects, such as sleep disturbance, depression, nausea, anxiety, fatigue, and vomiting [2]. The widespread use of a variety of nutritional, psychological and natural medical approaches, collectively-termed complementary and alternative medicine (CAM), has been well documented. It is defined as methods used in the diagnosis, treatment, or prevention of disease that complement mainstream medicine, as opposed to alternative therapies, which are used as a direct substitute for mainstream medicine [3]. The United States National Cancer Institute (NCI) has a long history demonstrating its interest in the scientific evaluation of complementary [4]. It supports CAM research which includes different methods and practices and other medical systems especially herb medicine [4]. Because of their extensive use and the therapeutic effects, there is an increasing interest and need to develop new methods for understanding of herb medicine, including the identification of active ingredients and their targets in the context of molecular network.

Over the past decade, the use of CAM therapies has been rapidly increasing among patients with cancer throughout the world [5]. A recent population-based survey of San Francisco women with breast cancer showed that 72% used at least one type of alternative modality 2 to 4 months after being diagnosed with breast cancer, especially in women with advanced disease [6]. Over the decades, researchers have developed lots of ways for the treatment of breast cancer, like surgery, hormonal therapy, adjuvant therapy, monoclonal antibodies, enzyme inhibitors and natural product therapy, et al. However, despite advances in screening, surgery, adjuvant radiation, and systemic therapy, as well as novel biologically targeted therapies, limitation still exists in their benefits, especially in advanced disease. Chemotherapy, radiation, endocrine therapy and biological agents may help, but with serious side effect and resistance against anticancer drugs they always result in low survival incidence and poor life quality.

To improve the quality and length of life when patients’ tumors have metastasized, nowadays, lots of researchers have dedicated themselves to searching for proper solutions from the resources of CAM, and indeed big progress has been made. The use of CAM has become increasingly popular in some fields, in which breast cancer is just one case for which herbal supplements are heavily promoted and easily accessible. As a whole medical system of CAM, Traditional Chinese Medicine (TCM) is derived from thousands of years of clinical application that has evolved independently from or parallel to allopathic conventional medicine and has been considered as one of the main items of the CAM [7].

As a matter of fact, TCM normally contains huge numbers of active pharmacological ingredients. Besides, herbal medicines also target several diverse genes or proteins to exert therapeutic effects, which make the investigation of their pharmacological molecular mechanisms extremely difficult. Although the concept of holistic medicine has been revealed to some extent in TCM practice, scientific evidence to illustrate the mechanisms of holistic medicine is severely lacking and many key problems associated still exist. Our previous work has firstly developed an integrated model of systems pharmacology to measure the efficacy of drugs, especially the multi-target drugs, and to reveal the functional mechanism of traditional medicine theories [3]. In recent years, many studies have shown that application of systems biology provides guidance to investigate the scientific connotation of TCM. The reason for this may be that on one hand, systems biology is generally recognized as a powerful tool to study complex life system that resembles the holism concept of channel tropism in TCM [8], and on the other hand, its applications to herbal medicine may provide new possibilities for investigating the explicit targets of medicinal herbs’ active ingredients and their interactions in the context of molecular networks, and thereby highlight the molecular mechanism of the herbs functions.

In this work, we proposed a systems-pharmacological model by combining oral bioavailability prediction, multiple drug-target prediction and validation, and network pharmacology techniques, to shed new lights on the effectiveness and mechanism of breast cancer-related TCMs. Specifically, we firstly explored 20 most frequently-used anti-breast cancer herbs and their corresponding constituents with a wide-scale text mining method. And since construction of an integrated model may be of great help for explaining the functions of herbal medicines and their relationships, we identified all candidate compounds of the herbs and their corresponding potential targets, and then mapped these compounds and targets onto functional ontologies to generate several drug-target networks to help understand the holistic and synergic essence of the herbal medicine from a systematic point of view.

## Materials and Methods

As herbs usually contain considerable chemical compounds, databases of each herb were built firstly. After that, a series of combinational integrated approaches were used to screen out active components from the databases, as well as to predict the potential targets. Finally, networks of compound-target and target-pathway were constructed based on the active components and predicted targets.

### Herbal medicines for breast cancer

With a text mining approach, we firstly extracted all available information about those herbs related with the treatment of breast cancer via the TCMSP database (Traditional Chinese Medicines Systems Pharmacology Database and Analysis Platform, http://lsp.nwsuaf.edu.cn/tcmspsearch.php). Since the degree of research of diverse herbs is different, presently, to lessen the possible bias of research extents and further evaluate the relationships between the herbs and depression, a parameter, i.e. the ratio of the number of anti-breast-herb-related articles/the number of herb-related articles is calculated. The p-value obtained from Eq 1 is applied to measure the significance of the relevance between each herb and breast cancer as reported literature (significant when p-value ≤ 0.01):.

$$P=1-\sum_{i=1}^{k-1}f(i)=1-\sum_{i=0}^{k-1}\frac{\left(\begin{array}{c} K\\ i \end{array}\right)\left(\begin{array}{c} N-K\\ n-i \end{array}\right)}{\left(\begin{array}{c} N\\ n \end{array}\right)}$$ (1)

where *N* is the total number of articles in PubMed and CNKI, *K* is the number of articles related to breast cancer, *n* the number of articles about one single herb and *k* is the number of articles about the effects of corresponding herbs on breast cancer, respectively [9]. In addition, more empirically based knowledge and TCM experience for the treatment of breast cancer are employed for the selection of herbs. Finally, a total of 20 herbs were collected. All the results are summarized in Table 1.

**Table 1:** Statistics and association analysis between 20 herbs and breast cancer.

English Name	Total (*n*)	Relevant to breast cancer (*k*)	*p*-value
Folium Artemisiae Argyi	52351	1114	p«0.01
Dysosmae Verspiellis Rhixoma Et Radix	4339	499	p«0.01
Atractylodes Macrocephala Koidz	365271	9673	p«0.01
Mylabris	14229	2419	p«0.01
Radix Salviae	481101	13666	p«0.01
Curcumae Rhizoma	73256	5610	p«0.01
Stephaniae Tetrandrae Radix	61204	3297	p«0.01
Curcumaelongae Rhizoma	81457	8266	p«0.01
Cortex Moutan	62681	2185	p«0.01
Caulis Akebiae	61689	1042	p«0.01
Eriobotryae Folium	18905	573	p«0.01
Pseudobulbus Cremastrae Seu Pleiones	16264	2294	p«0.01
Crataegi Folium	7699	377	p«0.01
Cornus Officinalis Sieb. Et Zucc	68476	2209	p«0.01
Asparagi Radix	95185	11382	p«0.01
Semiaquilegiae Radix	3301	269	p«0.01
Artemisiae Scopariae Herba	89803	2121	p«0.01
Gleditsiae Spina	20102	1125	p«0.01
Hedyotis diffusa	62308	4719	p«0.01
Ampelopsis japonica (Thunb.)Makino	4311	131	p«0.01

The total number of articles (N) in PubMed and CNKI is 104544921. The number of articles related to breast cancer (K) is 791592.

#### Chemical structures'construction

All ingredients of these 20 herbs were collected from (1) literature, (2) Chinese Academy of Sciences Chemistry Database (http://www.organchem.csdb.cn), and (3) Chinese Herbal Drug Database. Finally, a total of 1618 chemicals were collected. And all their chemical structures were either obtained from the Chemical Book (http://www.chemicalbook.com), NCBI PubChem database (http://www.ncbi.nlm.nih.gov/pccompound), or drawn by using ISIS Draw 2.5 (MDL Information Systems, Inc.) and further optimized by Sybyl 6.9 (Tripos Associates, St. Louis, MO). Since for oral-administrated medicine, a deglycosylation of the glucosides will appear in the intestinal tract by enteric bacteria, all compounds with glycosyl are further deglycosylated according to the rule of glycosidase hydrolysis reaction, and the resulted non-overlapping products are obtained and are further optimized according to those procedures described above [10].

### Oral bioavailability (OB) prediction

Oral route, the mainly drug delivery system, is commonly used in the administration of herbal medicines. So in the development of herbal drugs intended for oral use, good drug absorption and appropriate drug delivery are very important. OB, the percentage of an oral dose able to produce a pharmacological activity, is one of the most desirable attributes of a new drug. To calculate the OB, we have utilized a novel and robust in-house system OBioavail 1.1 integrating with the metabolism (P450 3A4) and transport (P-glycoprotein) information [11, 12]. Based on P450 3A4 docking score, the set of drug compounds were grouped into four subsets. Then the descriptors of the subsets were calculated by DRAGON professional (version 5.6; Talete SRL: Milano, Italian, 2006). High predictability was achieved by using Support Vector Machine (SVM), with the regression coefficients of internal training and external test data R_training_ = 0.89 and R_test_ = 0.85 and the standard errors of estimate SEE_training_ = 0.35, SEE_test_ = 0.42, respectively. We can discard the compounds with poor OB, thus may discover the bioactive compounds or lead compounds in herbal medicine for further development by this software.

The criteria used here are based upon careful consideration of the following: (1) information from the studied herbs can be extracted as much as possible using the least number of chemical ingredients; (2) the obtained model can be reasonably explained by the reported pharmacological data. In this work, compounds with OB ≥ 30% were selected as the candidate bioactive molecules.

### Drug-likeness (DL) evaluation

DL is a widely used concept in drug discovery for how “drug-like” a substance is with respect to factors which ultimately influences the absorption, distribution, metabolism, and excretion (ADME) of the substance in human body by affecting its pharmacodynamics and pharmacokinetics [13]. DL parameter between the compound structure and the drug molecular structure obtained from Drug Bank was evaluated by Tanimoto coefficient defined as:

$$F(A,B)=\frac{A*B}{{\left|A\right|}^{2}+{\left|B\right|}^{2}-A*B}$$

Here, *A* represents the new compound, and *B* represents the average drug-likeness index of all 6511 molecules in DrugBank database (access time: June 1st, 2011, http://www.drugbank.ca/). In this work, compounds with DL ≥ 0.18 were selected as the candidate bioactive molecules, due to that the average value of DL for all 6511 molecules in Drug Bank is 0.18. Only those ingredients who meet both the OB and DL screening criteria are finally treated as candidate compounds.

### Target prediction and validation

Previously in our work, we have predicted the potential targets of human and viral origins based on a systematic model [14], which efficiently integrated the chemical, genomic and pharmacological information for drug targeting and discovery on a large scale based on two powerful methods of Random Forest (RF) and Support Vector Machine (SVM). The sensitivity (SE) of the present models describes the ratio of correctly predicted interactions to the total number of the drug-target interactions, whereas the specificity (SP) refers to the ratio of correctly predicted non-interactions to the total number of the drug-target non-interactions. The integrated parameter concordance (the ratio of correctly predicted compound-protein pairs to the total number of tested compound-protein pairs, CO) gives an overall model performance value. This model shows an impressive performance of prediction for drug-target interaction, with a concordance of 86%, a sensitivity of 80% and a specificity of 93%, respectively. According to the possibilities of compounds interacting with candidate targets in the RF and SVM models, respectively, The proteins with the value predicted by both methods of RF and SVM greater than 0.8 and 0.7 respectively were chosen as potential targets (As displayed in the S1 Table).

### Network construction

The analysis of networks has gained extensive attentions in biological and even TCM fields, due to the recent explosion of publicly available high-throughput biological data. Such analysis also provides a unifying language to describe the relations within complex systems and to understand the physiological functions. To characterize the multicomponent therapeutic mechanisms of herbal medicine in the treatment of breast cancer from a network target perspective, we constructed two kinds of visualized networks: compound-target network (C-T network) and target-pathway network (T-P network), to get insight into new possibilities for understanding of the holistic, complementary and synergic essence of herbal medicine.

#### C-T network

All active substances in both the herbs and their targets were used to generate a bipartite graph of drug-protein interactions. In this graph, a drug and a protein were connected to each other when the protein was a target of the drug, and thus a C-T network was built. Not all compounds have relatively potential target. Since presently 20 compounds have no potential target, they were removed from the nets accordingly.

#### T-P network

The information about selected pathways was initially collected by the KEGG (Kyoto Encyclopedia of Genes and Genomes), and then the T-P network was built by connecting the targets and their related signaling pathways. Targets and their related signaling pathways were represented as nodes and their interactions as links, respectively, and all visualized networks were generated by Cytoscape 2.8.3, a standard tool for biological network visualization and data integration [15]. Finally, the quantitative properties of these networks were analyzed by two plugins including Network Analyzer.

## Results and Discussion

During the past few years, the pharmaceutical industry has seen a shift from the “1 disease, 1 target, 1 drug” and “1drug fits all” approaches to the pursuit of combination therapies that include more than one active ingredient [16]. It is believed that multiple components could hit multiple targets and exert synergistic therapeutic efficacies. In TCM, as one of the most important theories, “multiple herbal drugs for one disease” describes how different drugs treat the same disease, which implies that the drugs probably share common targets. The complexity of the chemical components makes it extremely difficult to understand the mechanisms of action for herbal medicines from a molecular or systems level. In the present study, novel systems pharmacology approach and network biology were applied to uncover the mechanism of the TCM rule from a molecular systematic level, in hope that it may also provide useful information for finding novel anti-breast-cancer leading compounds.

### Physicochemical property analysis

In order to analyze statistically the drug-like physicochemical properties of active compounds, herbal ingredient comparisons based on chemical properties are performed by considering 8 common molecular descriptors, which includes MW (molecular weight), nCIC (number of rings), nHDon (number of hydrogen-bond donor), nHAcc (number of hydrogen-bond acceptor), RBN (number of rotatable bonds), Hy (hydrophilic factor), TPSA (topological polar surface area) and MlogP (Moriguchi octanol-water partition coefficient), since these parameters reflect the basic characteristics of molecules including especially their pharmacokinetic properties [17]. In addition, these 8 molecular descriptors of all compounds from DrugBank are also calculated for a comparison to analyze the similarities or differences with our herbal ingredients in the property spaces. All molecular descriptors are calculated by DRAGON software in the present study.

**Fig 1:**
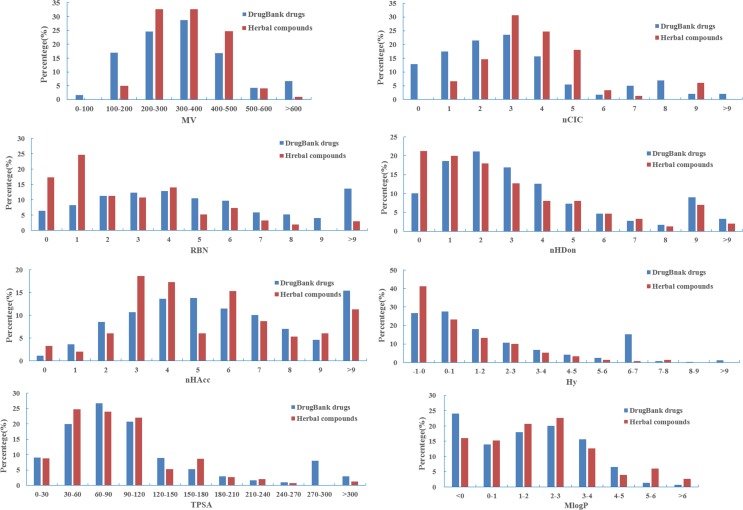
The profile distributions of eight important molecular properties for DrugBank drugs and herbal compounds.

Fig 1 depicts the distributions of selected properties among the herbal compounds and DrugBank drugs. The average values of these properties (mean and Standard Deviation) for these drugs in DrugBank as well as herbal compounds are displayed in Table 2. As shown in Fig 1, the molecular weight distribution for DrugBank drugs follows a Gaussian distribution, and the distribution characteristics are similar to those in the herbal compounds. In addition, herbal compounds exhibit an obvious narrower distribution than DrugBank from MW, nCIC and Hy descriptors, indicating that herbal compounds are less diverse than those of DrugBank drugs [18]. The calculated average values for molecular weight (see Table 2 and S2 Table) reflect the observations above. Because of the wider distribution in DrugBank drugs, the mean of molecular weight is higher for these compounds. In addition, the MlogP of herbal compounds (1.87**±**2.08) are larger than the ones of those DrugBank drugs (1.33**±**2.50), indicating that the molecules in herbal are more soluble in neutral solvents and much more hydrophobic than the DrugBank drugs. The nCIC, as a measurement for the rigidity of molecules, the average nCIC per molecule in herbal compounds (3.52**±**1.37) is larger than the DrugBank drugs (2.46**±**1.72). However, the average RBN per molecule of the herbal compounds (2.86**±**2.67) is lower than those of Drug-Bank ones (5.58**±**5.88). Thus, compounds in herbals probably have less thermodynamic advantages to achieve favorable binding properties than those in the DrugBank. Moreover, the nHDon and nHAcc of herbal compounds (2.57**±**2.47 and 5.63**±**3.50) has the lower donor/acceptor atoms for H-bonds than those (3.17**±**3.50 and 6.46**±**5.59) of DrugBank ones. Furthermore, the herbals compounds (90.15**±**57.95) have the lower average TPSA values than that of DrugBank compounds (99.93**±**90.43). All statistical analysis indicates two out of all eight descriptors between DrugBank drugs and herbal compounds are significantly different with p <0.01, including nCIC (2.46**±**1.72 versus 3.52**±**1.37) and RBN (5.58**±**5.88 versus 2.86**±**2.67).

**Table 2:** Comparison of molecular properties between herbal compounds and DrugBank drugs.

Index	MW (±SD)	nCIC (±SD)	RBN (±SD)	nHDon (±SD)	NHAcc (±SD)	Hy (±SD)	TPSA(Tot) (±SD	MlogP (±SD)
**DrugBank**	346.57 (208.89)	2.46 (1.72)	5.58 (5.88)	3.17 (3.50)	6.46 (5.59)	1.47 (2.76)	99.93 (90.43)	1.33 (2.50)
**Herbal**	344.15 (113.89)	3.52 (1.37)**	2.86 (2.67)**	2.57 (2.47)*	5.63 (3.50)	0.83 (1.80)	90.15 (57.95)	1.87 (2.08)*

*p < 0.05

**p < 0.01.

### OB prediction and DL calculation

For multi-compound medicinal herbs, it is usually quite difficult to distinguish those active ingredients from the compound pool of the TCMs due to the difficulties in current experimental methods, when virtual screening approaches are always of great help. In present work, a robust in-house system OBioavail 1.1 we developed previously was integrated with the metabolism information to predict a compound’s human oral bioavailability [19]. In addition, the drug-likeness property based on the Tanimoto coefficient was also applied to screen out those compounds which are deemed active in an herb. The model was built based on a set of 805 structurally diverse drug and drug-like molecules which have been critically evaluated for their human oral bioavailability. The multiple linear regression, partial least square and SVM methods were employed to build the models, resulting in an optimal model with R^2^ = 0.80, SEE = 0.31 for the training set, Q^2^ = 0.72, SEP = 0.22, respectively. At last, 150 candidate compounds were screened from the 1618 chemicals which possess not only satisfactory drug-likeness (DL>0.18), but also OB (>30%). As a result, a total of 150 bioactive ingredients of the 20 herbs are obtained (shown in Table 3). Here, in order to illuminate clearly, three representative herbal medicines are specified in detail to interpret these filtering principles.

**Table 3:** 150 bioactive compounds of the 20 herbs with their predicted OB and DL values.

**ID**	**Compound**	**OB**	**DL**	**Medicine**
AY01	B-sitosterol	36.91	0.75	Folium Artemisiae Argyi
AY02	Quercetin	3.15	0.28	Folium Artemisiae Argyi
AY03	Luteolin	13.36	0.25	Folium Artemisiae Argyi
AY04	Apigenin	46.23	0.34	Folium Artemisiae Argyi
AY05	Jaceosidin	17.26	0.38	Folium Artemisiae Argyi
AY06	Eupatilin	18.44	0.45	Folium Artemisiae Argyi
BJ01	Kaempferol	41.88	0.24	Dysosmae Verspiellis Rhixoma Et Radix
BJ02	Quercitrin	4.04	0.74	Dysosmae Verspiellis Rhixoma Et Radix
BJ03	Beta-sitosterol	36.91	0.75	Dysosmae Verspiellis Rhixoma Et Radix
BJ04	4'-demethylpodophyllotoxin	27.05	0.82	Dysosmae Verspiellis Rhixoma Et Radix
BJ05	Deoxypodophyllotoxin	37.75	0.83	Dysosmae Verspiellis Rhixoma Et Radix
BJ06	Picropodophyllin	51.77	0.86	Dysosmae Verspiellis Rhixoma Et Radix
BJ07	Podophyllotoxin	59.94	0.86	Dysosmae Verspiellis Rhixoma Et Radix
BJ08	Podophyllotoxone	49.61	0.86	Dysosmae Verspiellis Rhixoma Et Radix
BZ01	Atractylenolide I	37.37	0.15	Atractylodes Macrocephala Koidz
BZ02	AtractylenolideII	47.5	0.15	Atractylodes Macrocephala Koidz
BZ03	Atractylenolide III	68.11	0.18	Atractylodes Macrocephala Koidz
BZ04	8β-ethoxy atractylenolide III	35.95	0.21	Atractylodes Macrocephala Koidz
BZ05	3β-acetoxyatractylone	54.07	0.22	Atractylodes Macrocephala Koidz
BM01	Norcantharidin	97.71	0.07	Mylabris
BM02	Cantharidin	51.23	0.1	Mylabris
DS01	Digallate	61.85	0.26	Radix Salviae
DS02	Dehydrotanshinone II A	43.76	0.4	Radix Salviae
DS03	2-(4-hydroxy-3-methoxyphenyl)-5-(3-hydroxypropyl)-7-methoxy-3-benzofurancarboxaldehyde	62.78	0.4	Radix Salviae
DS04	Formyltanshinone	73.44	0.42	Radix Salviae
DS05	Methylene tanshinquinone	37.07	0.36	Radix Salviae
DS06	Przewalskin B	110.32	0.44	Radix Salviae
DS07	Przewaquinone B	62.24	0.41	Radix Salviae
DS08	Przewaquinone C	55.74	0.4	Radix Salviae
DS09	Tanshinaldehyde	52.47	0.45	Radix Salviae
DS10	Danshenol B	57.95	0.56	Radix Salviae
DS11	Danshenol A	56.97	0.52	Radix Salviae
**ID**	**Compound**	**OB**	**DL**	**Medicine**
DS12	Cryptotanshinone	52.34	0.4	Radix Salviae
DS13	Danshenspiroketallactone	50.43	0.31	Radix Salviae
DS14	Deoxyneocryptotanshinone	49.4	0.29	Radix Salviae
DS15	Dihydroisotanshinone I	20.91	0.36	Radix Salviae
DS16	Dihydrotanshinone I	45.04	0.36	Radix Salviae
DS17	Epidanshenspiroketallactone	68.27	0.31	Radix Salviae
DS18	Isocryptotanshi-none	54.98	0.39	Radix Salviae
DS19	Isotanshinone IIB	21.07	0.45	Radix Salviae
DS20	Isotanshinone II	49.92	0.4	Radix Salviae
DS21	Isotanshinone I	29.72	0.36	Radix Salviae
DS22	Miltionone II	71.03	0.44	Radix Salviae
DS23	Neocryptotanshinone II	39.46	0.23	Radix Salviae
DS24	Neocryptotanshinone	52.49	0.32	Radix Salviae
DS25	Prolithospermic acid	64.37	0.31	Radix Salviae
DS26	(2R)-3-(3,4-dihydroxyphenyl)-2-[(Z)-3-(3,4-dihydroxyphenyl)acryloyl]oxy-propionic acid	109.38	0.35	Radix Salviae
DS27	(Z)-3-[2-[(E)-2-(3,4-dihydroxyphenyl)vinyl]-3,4-dihydroxy-phenyl]acrylic acid	88.54	0.26	Radix Salviae
DS28	(6S)-6-hydroxy-1-methyl-6-methylol-8,9-dihydro-7H-naphtho[8,7-g]benzofuran-10,11-quinone	75.39	0.46	Radix Salviae
DS29	Tanshinone IIA	49.89	0.4	Radix Salviae
DS30	(6S)-6-(hydroxymethyl)-1,6-dimethyl-8,9-dihydro-7H-naphtho[8,7-g]benzofuran-10,11-dione	65.26	0.45	Radix Salviae
DS31	Tanshinone VI	45.64	0.3	Radix Salviae
DS32	Tanshinone I	29.27	0.36	Radix Salviae
WZ01	γ-elemene	23.79	0.06	Curcumae Rhizoma
WZ02	Beta-elemene	25.63	0.06	Curcumae Rhizoma
WZ03	δ-elemene	21.47	0.06	Curcumae Rhizoma
WZ04	(1R,10R)-epoxy-1,10-dihydrocurdione	36.73	0.12	Curcumae Rhizoma
WZ05	Isocurcumenol	97.67	0.13	Curcumae Rhizoma
WZ06	Bisdemethoxycurcumin	77.38	0.26	Curcumae Rhizoma
WZ07	Curcumenol	96.72	0.08	Curcumae Rhizoma
WZ08	Curdione	35.05	0.11	Curcumae Rhizoma
**No.**	**Compound**	**OB**	**DL**	**Medicine**
WZ09	Curzerenone	52.68	0.13	Curcumae Rhizoma
WZ10	Isocurcumol	101.1	0.08	Curcumae Rhizoma
FJ01	Tetrandrine	26.64	0.1	Stephaniae Tetrandrae Radix
FJ02	Hesperetin	70.31	0.27	Stephaniae Tetrandrae Radix
JH01	(+)-alpha-curcumene	26.56	0.06	Curcumae longae Rhizoma
JH02	Alpha-curcumene	4.68	0.06	Curcumae longae Rhizoma
JH03	Procurcumadiol	69.82	0.13	Curcumae longae Rhizoma
JH04	Curcumol	103.55	0.13	Curcumae longae Rhizoma
JH05	Bisdemethoxycurcumin	3.55	0.26	Curcumae longae Rhizoma
JH06	Demethoxycurcumin	4.37	0.33	Curcumae longae Rhizoma
JH07	Dihydrocurcumin	5.91	0.41	Curcumae longae Rhizoma
JH08	Curcumin	4.37	0.04	Curcumae longae Rhizoma
MD01	Gallate acid	25.01	0.79	Cortex Moutan
MD02	Paeoniflorin	19.92	0.78	Cortex Moutan
MD03	Mairin	55.38	0.78	Cortex Moutan
MD04	(+)-catechin	54.83	0.24	Cortex Moutan
MD05	Paeonol	28.79	0.04	Cortex Moutan
MD06	Paeoniflorin_qt	68.18	0.4	Cortex Moutan
MD07	Oxypaeoniflorin_qt	19.4	0.44	Cortex Moutan
MD08	Benzoyl paeoniflorin	31.14	0.54	Cortex Moutan
MD09	Oxypaeoniflorin	12.98	0.78	Cortex Moutan
MD10	4-O-methylpaeoniflorin_qt	67.24	0.43	Cortex Moutan
MD11	Paeonidanin_qt	65.31	0.35	Cortex Moutan
MT01	Ariskanina	109.51	0.4	Caulis Akebiae
MT02	Aristolochic acid	62.71	0.55	Caulis Akebiae
MT03	Hederagenin	36.91	0.75	Caulis Akebiae
MT04	Oleanolic acid	15.32	0.74	Caulis Akebiae
SZ01	Quercetin	46.43	0.28	Crataegi Folium
SZ02	Rutin	3.2	0.68	Crataegi Folium
SZ03	Vitexin	3.05	0.71	Crataegi Folium
SZ04	Hyperin	6.94	0.77	Crataegi Folium
SZ05	Vitexin-2-o-rhamnoside	6.98	0.8	Crataegi Folium
PP01	Corosolic acid	14.97	0.76	Eriobotryae Folium
PP02	Oleanolic acid	29.02	0.76	Eriobotryae Folium
PP03	Ursolic acid	16.77	0.75	Eriobotryae Folium
PP04	Maslinic acid	15.54	0.74	Eriobotryae Folium
**No.**	**Compound**	**OB**	**DL**	**Medicine**
PP05	(2R,3R,10S)-2,10-bis(3,4-dihydroxyphenyl)-3,5-dihydroxy-3,4,9,10-tetrahydro-2H-pyrano[6,5-h]chromen-8-one	65.26	0.93	Eriobotryae Folium
PP06	(4R,8R,9R)-4,8-bis(3,4-dihydroxyphenyl)-5,9-dihydroxy-4,8,9,10-tetrahydro-3H-pyrano[6,5-h]chromen-2-one	58.16	0.93	Eriobotryae Folium
PP07	(2R,3R,4S)-2-(3,4-dihydroxyphenyl)-4-(2,4,6-trihydroxyphenyl)chroman-3,5,7-triol	72.41	0.64	Eriobotryae Folium
SC01	Cirrhopetalanthrin	24.68	0.93	Pseudobulbus Cremastrae Seu Pleiones
SC02	Shanciol	5.44	0.92	Pseudobulbus Cremastrae Seu Pleiones
SC03	Shanciol E	15.62	0.92	Pseudobulbus Cremastrae Seu Pleiones
SC04	Shanciol F	17.49	0.11	Pseudobulbus Cremastrae Seu Pleiones
SC05	Cremastrine	17.3	0.59	Pseudobulbus Cremastrae Seu Pleiones
SC06	Sanjidin A	3.82	0.59	Pseudobulbus Cremastrae Seu Pleiones
SC07	Sanjidin B	3.82	0.53	Pseudobulbus Cremastrae Seu Pleiones
SC08	Pleionin A	35.68	0.65	Pseudobulbus Cremastrae Seu Pleiones
SC09	Pleionol	41.98	0.04	Pseudobulbus Cremastrae Seu Pleiones
SY01	Gallic acid	25.01	0.44	Cornus Officinalis Sieb. Et Zucc
SY02	Loganin	16.8	0.5	Cornus Officinalis Sieb. Et Zucc
SY03	Morroniside	13.86	0.04	Cornus Officinalis Sieb. Et Zucc
SY04	Protocatechuic acid	25.47	0.09	Cornus Officinalis Sieb. Et Zucc
SY05	Swertiamarin_qt	2.58	0.09	Cornus Officinalis Sieb. Et Zucc
SY06	Swertiamarin	21.9	0.42	Cornus Officinalis Sieb. Et Zucc
SY07	Malkangunin	57.71	0.63	Cornus Officinalis Sieb. Et Zucc
SY08	Telocinobufagin	69.99	0.79	Cornus Officinalis Sieb. Et Zucc
SY09	Cornuside	2.61	0.71	Cornus Officinalis Sieb. Et Zucc
SY10	Cornuside_qt	2.37	0.39	Cornus Officinalis Sieb. Et Zucc
SY11	Gemin D	68.83	0.56	Cornus Officinalis Sieb. Et Zucc
TD01	Asparaside A	9.48	0.02	Asparagi Radix
TD02	Aspafilioside A_qt	17.66	0.81	Asparagi Radix
TD03	Diosgenin	80.88	0.81	Asparagi Radix
TD04	Asparaside a_qt	30.6	0.86	Asparagi Radix
TK01	Griffonilide	43.15	0.05	Semiaquilegiae Radix
TK02	Berberrubine	35.74	0.73	Semiaquilegiae Radix
TK03	Thalifendine	44.41	0.73	Semiaquilegiae Radix
**No.**	**Compound**	**OB**	**DL**	**Medicine**
YC01	Capillarin	87.01	0.08	Artemisiae Scopariae Herba
YC02	Artepillin A	68.32	0.24	Artemisiae Scopariae Herba
YC03	Chlorogenic acid	13.99	0.41	Artemisiae Scopariae Herba
YC04	Areapillin	48.96	0.41	Artemisiae Scopariae Herba
YC05	Isoarcapillin	57.4	0.41	Artemisiae Scopariae Herba
YC06	Capillarisin	57.56	0.31	Artemisiae Scopariae Herba
YC07	4'-methylcapillarisin	72.18	0.35	Artemisiae Scopariae Herba
YC08	Demethoxycapillarisin	52.33	0.25	Artemisiae Scopariae Herba
ZJ01	Quercetin	46.43	0.28	Gleditsiae Spina
ZJ02	(-)-taxifolin	60.51	0.27	Gleditsiae Spina
ZJ03	Fisetin	52.6	0.24	Gleditsiae Spina
ZJ04	Fustin	50.91	0.24	Gleditsiae Spina
ZJ05	3β-acetoxyolean-12-en-28-oic acid	40.21	0.4	Gleditsiae Spina
BH01	Deacetyl asperulosidic acid	3.24	0.45	Hedyotis Diffusa
BH02	Deacetyl asperulosidic acid _qt	30.29	0.1	Hedyotis Diffusa
BH03	Deacetyl asperulosidic acid methyl ester	4.29	0.48	Hedyotis Diffusa
BH04	Deacetyl asperuloside acid_qt	62.46	0.11	Hedyotis Diffusa
BL01	Cis-resveratrol	41.13	0.11	Ampelopsis Japonica (Thunb.) Makino
BL02	Oleanolic acid	29.02	0.76	Ampelopsis Japonica (Thunb.) Makino
BL03	(2R,3R,4S)-4-(4-hydroxy-3-methoxy-phenyl)-7-methoxy-2,3-dimethylol-tetralin-6-ol	66.51	0.39	Ampelopsis Japonica (Thunb.) Makino
BL04	(+)-catechin	54.83	0.24	Ampelopsis Japonica (Thunb.) Makino
BL05	Digallate	61.85	0.26	Ampelopsis Japonica (Thunb.) Makino
BL06	(-)-catechin gallate	53.57	0.75	Ampelopsis Japonica (Thunb.) Makino

#### Radix salvia

*Radix Salviae*, derived from the dried root or rhizome of Salvia miltiorrhiza Bge (Chinese Pharmacopoeia Committee, 2005), is one of the oldest and most frequently used TCMs. It was recorded in *The China Pharmacopoeia* with the Chinese name *Danshen* and has been widely used for the treatment of various kinds of diseases [20]. Various *Fufang Danshen* prescriptions are commercially available, such as *Fufang Danshen* tablet, *Guanxin Danshen* tablet, Compound *Danshen* dripping pill and *Danqi* tablet [21]. Pharmacological research has revealed that these preparations have the effects of activating blood circulation, dilating coronary artery and antagonizing myocardial ischemia, and potent therapeutic effects have been demonstrated for treating coronary heart disease, cardiac angina and atherosclerosis in the clinic [22]. It also has been widely used for the treatment of menstrual disorders, menorrhealgia, insomnia, menostasis, blood circulation diseases, and other cardiovascular diseases [22].

The major bioactive constituents of *Radix Salviae* can be classified into hydrophilic depsides derived from caffeic acid such as salvianolic acids and lipophilic components including diterpenoids tanshinones [19]. In this work, 174 compounds of various types have been identified in *Radix Salviae*, out of which 28 molecules demonstrate good OB and DL. It is interesting that among all the 28 compounds, some were already reported to be pharmacologically effective for the treatment of various symptoms. For instance, tanshinone IIA has been confirmed to have anti-inflammatory, anti-ischemic, antioxidant, and antitumor activities [23]. Tanshinone IIA not only has a powerful inhibitory effect on the proliferation of ER-positive human breast cells *in vitro*, but also significantly inhibits the growth of human ER-negative breast *in vivo*, without any untoward toxicity. The anticancer activity of tanshinone IIA could be attributed in part to its inhibition of proliferation and apoptosis induction of cancer cells through cell proliferation, apoptosis, signal transduction, transcriptional regulation, angiogenesis, invasive potential and metastatic potential of cancer cells [24].

Besides the above 28 molecules, 4 compounds, though with low OB or DL values, are also deemed as active components due to their reported therapeutical effects, like tanshinone I that actually significantly induced the apoptosis in MCF-7 and MDA-MB-231 human breast cancer cells. Otherwise, the activation of caspase 3, anti-apoptotic protein Bcl-2, pro-apoptotic protein Bax have been reported to mediate the induction of apoptotic cell death [25]. Owing to the profound pharmacological effects, these compounds are also selected for further research as well. In brief, 32 ingredients from *Radix Salviae* are assumed playing major roles in the treatment of breast cancer, and thus chosen as active compounds for further study.

#### Atractylodes macrocephala koidz

*Atractylodes Macrocephala Koidz* (Bai-Zhu), as a valuable traditional Chinese medicine, is an important component of several Chinese herbal prescriptions and has been used for treating various diseases for thousands of years all over the world duo to its special pharmacological activities [23]. *Atractylodes Macrocephala Koidz*, a member of the Compositae can invigorate the spleen, and cure patients with splenic asthenia, anorexia, oedema, excessive perspiration and abnormal fetal movement. The rhizomes of *Atractylodes Macrocephala Koidz* have also shown a variety of other pharmacological activities such anti-inflammatory, antioxidant and as antitumor [26].

The chemical investigations on the rhizomes of *Atractylodes Macrocephala Koidz* have demonstrated the anti-tumor and anti-inflammatory activities of the main active constituents of the herb [27]. In these ingredients, eudesmane-type sesquiterpenoids, such as atractylenolides I, II, III, etc. are the major ones which exhibit the activity of gastro protective, anti-inflammatory and anti-tumor properties [28, 29]. Notably, atractylenolides I could reduce the production of pro-inflammatory cytokines, such as TNF-α, IL-1β, IL-6 [30]. In addition, at a concentration of 10 μM, atractylenolide I, atractylenolide II and atractylenolide III also had inhibition ratios of 94.56 ± 0.70%, 90.93 ± 1.41% and 86.31 ± 8.46%, respectively, which indicates that atractylenolide and its derivatives may serve as potential aromatase inhibitors for the treatment of breast cancer [27].

#### Hedyotis diffusa

*Hedyotis diffusa*, belonging to the Rubiaceae family, is a medicinal herb widely distributed in Northeast Asia. As a well-known traditional Chinese folk-medicine, *Hedyotis diffusa* has long been used as a major component in many TCM formulas for the clinical treatment of hepatitis, tonsillitis, sore throat, appendicitis, urethral infection, and malignant tumors of the liver, lung, and stomach [31]. It is reported to possess various pharmacological activities such as anti-cancerous, anti-oxidative, anti-inflammatory, hepato-protective, and neuroprotective activities [32]. *Hedyotis diffusa* has also been used for treating many types of cancer and tumor in Taiwan and China for a long time [32]. *Hedyotis diffusa* has been demonstrated significant anti-proliferative effects and apoptotic responses by inducing p53 gene expression in ERα-positive MCF-7 breast cancer cells. Furthermore, *Hedyotis diffusa* inhibits anchorage-dependent and-independent cell growth in ERα-positive breast cancer cells in a dose-related manner [33].

In this study, 4 components from *Hedyotis diffusa* which include deacetyl asperulosidic acid and its derivatives demonstrate good bioavailability, and thus were chosen as candidate components. It is reported that asperulosidic acid has numerous pharmacological activities including anti-inflammatory, anticancer and hepatoprotective effects [34]. They have an inhibitory effect on tumor cell proliferation through cell cycle arrest [35]. The mechanism of anti-tumor activity is that they have effects on the inhibition of cell proliferation and stimulation of cell apoptosis in human cancer cells [34]. In addition, deacetyl asperulosidic acid methyl ester and deacetyl asperulosidic acid have the activity of analgesia which reduces the risk of cancer recurrence [36, 37].

To sum it up, 150 ingredients of all these 20 herbs are finally screened and regarded as candidate compound.

### Drug targeting and validating for the therapy of breast cancer

Identification of the protein targets of a bioactive small molecule is the most important step in new drug development because small molecules typically exert their bioactive effects through interactions with proteins. The interactions between the small molecule and its target protein are the key to understanding the small molecule’s cellular mechanism. Traditionally, expressed-sequence tags (ESTs), serial analysis of gene expression (SAGE), homology cloning and relevant approaches are adopted to confirm the therapeutic targets of active chemicals [38]. However, given the time-consuming, expensive, challenging feature and a narrow application scope of this process, computational methods always become the first choice. In this section, in order to probe the binding of Chinese herbs to their targets in breast cancer, we developed a simple, robust, unbiased and universally applicable target identification approach on the basis of the RF and SVM techniques. It combines the chemical, genomic and pharmacological information for drug targeting and discovery on a large scale.

As mentioned above, 150 compounds were screened out. However, the way how they interact with their specific acting targets still remains unknown. Based on the application of RF and SVM, a total of 83 proteins were identified as targets to interact with those candidate compounds of the 20 herbs. Among these, finally 33 targets are chosen which are highly associated with breast cancer, such as hormonal receptors, inflammatory mediators. Further analysis about the connections between the drugs and these targets are referred in network construction and analysis. The detailed information of the 33 target proteins is described in Table 4.

**Table 4:** The information of breast cancer targets.

Protein Name	Gene Name	UniProt ID
Muscarinic acetylcholine receptor M4	CHRM4	P08173
Muscarinic acetylcholine receptor M5	CHRM5	P08912
Alpha-1A adrenergic receptor	ADRA1A	P35348
Alpha-1B adrenergic receptor	ADRA1B	P35368
Calcium-activated potassium channel subunit alpha-1	KCNMA1	Q12791
Mu-type opioid receptor	OPRM1	P35372
Progesterone receptor	PGR	P06401
Muscarinic acetylcholine receptor M3	CHRM3	P20309
Muscarinic acetylcholine receptor M2	CHRM2	P08172
Muscarinic acetylcholine receptor M1	CHRM1	P11229
Retinoic acid receptor RXR-alpha	RXRA	P19793
DNA topoisomerase 2-alpha	TOP2A	P11388
Carbonic anhydrase 2	CA2	P00918
Prostaglandin G/H synthase 1	PTGS1	P23219
Prostaglandin G/H synthase 2	PTGS2	P35354
Androgen receptor	AR	P10275
Nitric oxide synthase, inducible	NOS2	P35228
Nitric oxide synthase, endothelial	NOS3	P29474
Estrogen receptor	ESR1	P03372
Estrogen receptor beta	ESR2	Q92731
Serine/threonine-protein kinase Chk1	CHEK1	O14757
Serine/threonine-protein kinase pim-1	PIM1	P11309
Alpha-1D adrenergic receptor	ADRA1D	P25100
Alpha-2A adrenergic receptor	ADRA2A	P08913
Alpha-2B adrenergic receptor	ADRA2B	P18089
Alpha-2C adrenergic receptor	ADRA2C	P18825
Delta-type opioid receptor	OPRD1	P41143
Coagulation factor X	F10	P00742
Glucocorticoid receptor	NR3C1	P04150
Vascular endothelial growth factor receptor 2	KDR	P35968
Sodium-dependent dopamine transporter	SLC6A3	Q01959
Sodium-dependent noradrenaline transporter	SLC6A2	P23975
Sodium-dependent serotonin transporter	SLC6A4	P31645

### GOBP enrichment analysis for targets

For further exploring the functional annotation of our 33 potential targets of active compounds, Gene Ontology (GO) enrichment analysis was performed by linking the targets to DAVID (The Database for Annotation, Visualization and Integrated Discovery, http://david.abcc.ncifcrf.gov) bioinformatics resources to systematically analyzing their biological process. One of the three broad GO categories (the other two being “Molecular Function” and “Cellular Component”) Biological Process” (GOBP) was utilized to symbol gene function. Only GO terms with p-value ≤ 0.05 were selected.

**Fig 2:**
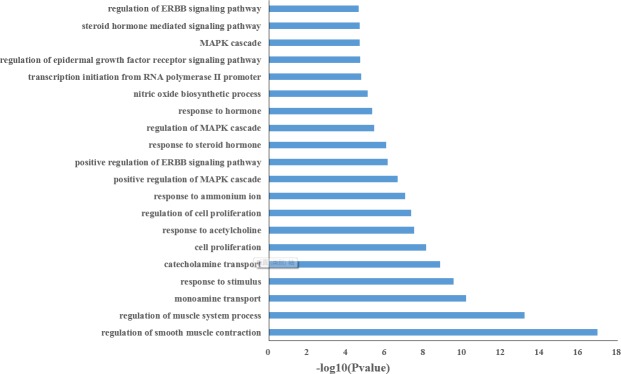
Gene Ontology (GO) analysis of therapy target genes. The y-axis shows significantly enriched ‘Biological Process ‘ (BP) categories in GO relative to the target genes, and the x-axis depicts the enrichment scores of these terms (*p*-value≤0.05).

Fig 2 and S3 Table list the top 20 significantly enriched GO terms, the results show that the majority of 33 targets are strongly associated with various biological processes by cluster analysis. As shown in Fig 2, it is interesting to note that a larger number of targets involve in inflammatory and hormones associated with a variety of biological processes such as regulation of MAPK cascade, response to steroid hormone, steroid hormone mediated signaling pathway, cell proliferation, which are closely related to the pathogenesis of breast cancer. For example, a hormone receptor estrogen is widely recognized for its role in breast cancer and the presence of estrogen receptor is of outstanding importance for treating breast cancer [39]. In addition, androgen receptor, another hormone receptor, it is a predictive factor for response to endocrine treatment in patients with breast cancer [40]. These results suggest that the active compounds of herbal medicines might exert the therapeutic effects on breast cancer through regulating the biological processes of their protein targets.

### Network construction and analysis

**Fig 3:**
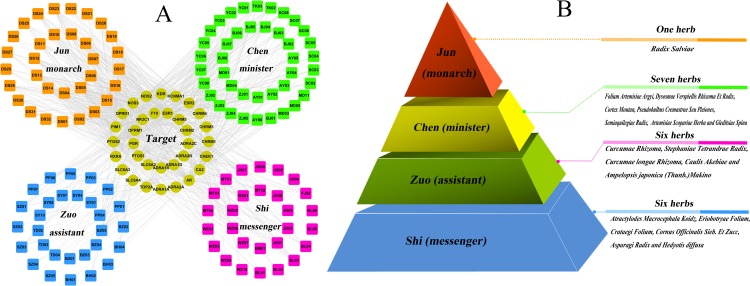
The global view of C-T network for the 20 breast cancer-related herbs. (A) 32 bioactive compounds (orange squares) from *Radix Salviae* applied as monarch herbal medicine play principal roles in therapeutic effect. 41 bioactive compounds (green squares) from 7 herbs represent those minister herbal medicine which increase the effects of *Radix Salviae*. 57 bioactive compounds (magenta and blue squares) from 12 herbs serve as assistant and messenger drugs, respectively. The yellow circles represent the target proteins of the active compounds. (B) The combination principle of Jun-Chen-Zuo-Shi.

With the growing understanding of the breast cancer, the focus of analyzing its treatment has shifted from the well-accepted ‘one target, one drug’ model design toward a new ‘multi-target, multi-drug’ model aimed at systemically modulating multiple targets in body. In the following part, the network pharmacology approaches are applied in the investigations. Based on these outcomes and to uncover the synergistic effects, our aim is to assess the mechanism and relationship between breast cancer and these targets by using compound-target (C-T) network analysis. As mentioned above, 33 predicted targets are screened out, which are targeted by 150 candidate components. After deleting all those compounds (altogether 20) with no targets, the resultant 130 candidate compounds and all their candidate targets are used to generate a graph of C-T interactions. As illustrated in Fig 3, the C-T network is constructed by all the active ingredients of 20 herbs. The squares and circles represent the potential compounds and targets, respectively.

#### The reasonability of the combination rule of Jun-Chen-Zuo-Shi

Traditional Chinese prescriptions organically combine various herbal drugs based on a certain composition principle. According to the traditional Chinese medicine theory, a TCM formula contains multiple interactive herbs. It is customary to rank the compositions into four categories, namely, Jun (monarch), Chen (minister), Zuo (assistant) and Shi (guide) with proper herbs to synergize the desirable effects and to minimize side effects integrally when analyzing the roles they play in the TCM formula. The monarch drug is an essential ingredient in a formula, which plays leading pharmacological effects aiming at the cause or the main syndrome of a disease. The minister drug helps strengthen the curative effect of the monarch drug, or, is used for treatment of accompanying symptoms [41]. The assistant drug is used to promote the treatment efficacy of the minister drug on chief syndromes/disease, or to aim at minor symptoms [42]. The Guide drug is to lead the ‘herbs’ impact to the disease location and to modulate the interaction among the herbs [43]. Various drugs in a prescription have their respective importance of effect in the order of ‘monarch, minister, assistant and messenger. The principles of “monarch”, “minister”, “assistant” and “messenger” signify the fact that each herbal ingredient of the recipe has a specific function within the composition, and is organized and arranged integrally under this principle to generate its specific effect [43].

The present work shows that the Jun herb possesses two extraordinary characteristics: many active molecules and powerful pharmacological effects. As shown in Fig 3, 32 ingredients from *Radix Salviae* which contain the major bioactive compounds in the 20 herbs are assumed playing major roles in the treatment of breast cancer. In order to quantify the polypharmacological effect, we count the number of targets for each drug, that is, the degree for each drug node in the drug-target network. As a result, *Radix Salviae* has the largest number of degree in the 20 herbs. Network analysis also shows that the average number of targets per compound is 12. All these 32 compounds are linked with more than two targets, proving the polypharmacology of this herb. For example, *Radix Salviae* has long time ago been used for treating atherosclerosis, cardiovascular diseases, stroke, inflammatory diseases and cancers. Its major bioactive constituents are multi-target drugs, whose molecular targets include transcription factors, scavenger receptors, ion channels, kinases, pro- and anti-apoptotic proteins, growth factors, inflammatory mediators, microRNA, and others [44]. This good hit rate indicates the rationality and reliability to find active compounds by using network-based analytical methods. Thus, it is assumed that *Radix Salviae* as monarch herbal medicine plays a principal role in therapeutic effect.

In this work, by compound structural comparisons and target analysis, to certain extent, 7 herbs include 41 ingredients contain about half of whole bioactive compounds. In addition, these herbs have the middle number of degree in the 20 herbs; the average number of potential targets per candidate compound is 7.24, and these compounds have many overlapping targets. This indicates that these 7 herbs and *Radix Salviae* could produce enhancing pharmacological synergism, due to that these herbs direct at a similar receptor target or physiological system. Thus, 7 herbs are assumed as minister herbal medicine increasing the effect of *Radix Salviae*.

The rest herbs (Fig 3) serve as assistant and messenger drugs which have the lowest number of active ingredients and also target comparatively less proteins displayed. The average number of potential targets per candidate compound is only 6.74. However, despite the small number of the active compounds and low degree, these herbs may have broad pharmacological actions with Jun/Chen herb, which was used to improve the pharmacological activity of monarch drug and ministerial drug. For example, *Curcumae Rhizoma* is an important TCM with ‘‘Huoxuehuayu” (promoting blood circulation and removing blood stasis) activity, and its essential oils contribute to several of its pharmacological effects [45]. It exerts synergistic antitumor activity with other herbs and thus *Curcumae Rhizoma* can increase the function of the herb combination [46]. Beta-*elemene* is the main components of essential oils in *Curcumae Rhizoma*. Beta-*elemene* -induced alteration of cell membrane permeability, which potentially results in the enhanced cellular uptake of taxanes, may contribute to the synergistic interactions of the combination treatment [47]. Beta-*elemene* increases anti-cancer in by combination with etoposide, which is mediated by the cleavage of poly (ADP-ribose) polymerase [47]. Therefore, *Curcumae Rhizoma* demonstrates the potentially beneficial clinical combination with other drugs. *Curcumae longae Rhizoma*extracts show a high anti-inflammatory effect after parenteral application in standard animal models of inflammation [48]. Its main activity component Curcumin has been shown to be synergistic with chemotherapy such as 5-fluorouracil, oxaliplatin, and gemcitabine in inhibiting tumor cell growth [49]. This synergistic effect appears to be partly due to the inhibition of NF-κB and growth factor receptors, which indicates that *Curcumae longae Rhizoma* might affect the pharmacological effects of other herbs due to the drug-drug interactions.

Taken together, these 20 herbs work together harmoniously to achieve an ideal therapeutic effect. All these results also explain why this formula takes the ‘‘Jun-Chen-Zuo-Shi” as the rule of prescription.

#### Illustrating the mechanism of action on treating breast cancer based on the C-T interactions

**Fig 4:**
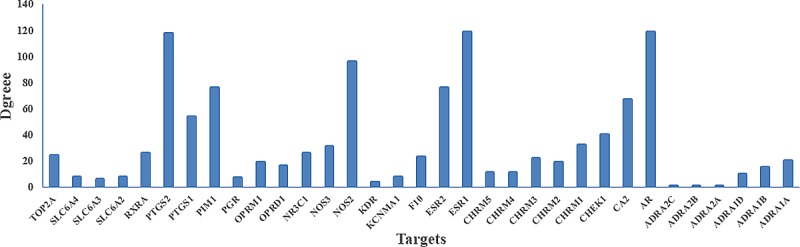
Distribution of the target proteins versus the drug node degree in the drug-target network.

Hormones play a critical role in breast carcinogenesis [50]. The sex hormones control the activation of responsive genes by first binding to specific receptors and forming complexes that can in turn bind to sequences in the promoters of downstream, hormone-responsive receptors, and thus steroid hormone receptors are candidates for breast cancer susceptibility receptors [51]. These hormonal receptors, namely, androgen receptor, glucocorticoid receptor, estrogen receptor, and progesterone receptor, have been widely targeted for developing treatments for breast cancer. As shown in Fig 4, several targets belong to the estrogen receptor-like family that displays the highest degree, such as ESR1 (estrogen receptor-α, degree = 119), ESR2 (estrogen receptor-β, degree = 76), AR (androgen receptor, degree = 119), NR3C1 (glucocorticoid receptor, degree = 26) and PGR (progesterone receptor, degree = 7), which are major therapeutic targets of breast cancer due to their dominant positions in this net.

For instance, (1) estrogen is widely recognized for its role in breast cancer; elevated urinary levels of estrogens is associated with an increased risk of breast cancer [52]. Knowledge of the central role of estrogen receptor in breast cancer has already led to the development of new preventive and therapeutic interventions that block receptor function or drastically reduce the levels of endogenous estrogen through the inhibition of its synthesis [52]. Additionally, endocrine therapies that target the ER are the cornerstone of breast cancer treatment for the majority of patients [39]. (2) AR, a member of the steroid receptor subfamily with well-known biological and therapeutic importance has been implicated in breast cancer pathogenesis [40]. AR may be both a prognostic factor for survival and a predictive factor for response to endocrine treatment in patients with breast cancer [53]. In addition, AR is expressed in normal breast epithelial cells and in approximately 70% to 90% of invasive breast carcinomas [40]. (3) PGR is ligand-activated transcription factor member of the steroid hormone family of nuclear receptor [54]. Progesterone is also critically involved in the development of the mammary gland and breast cancer, and its effects are mostly mediated via the progesterone receptor [55]. Progestational agents are widely used for oral contraception, menopausal hormone replacement therapy, and for treating breast cancer [54]. As a result, PGR is an important therapeutic target in breast cancer. (4) GR is a classic nuclear hormone receptor that dimerizes after ligand binding, translocates to the nucleus, and modulates the expression of many target genes [56]. NR3C1 activation has been demonstrated an inhibition capacity to apoptosis in breast epithelial cells, which play an important role in breast epithelial and breast cancer cell biology [57]. NR3C1 directly mediates the gene expression pathways associated with the aggressive behavior and early relapse in the breast cancer subtype. NR3C1-induced MAPK phosphatase-1 (MPK-1) expression inhibits the paclitaxel-associated MAPK activation and contributes to breast cancer cell survival [57]. Thus, NR3C1 expression may represent a novel therapeutic target for breast cancer. These results confirm the reliability of our network and these high-degreed targets are major therapeutic targets in the treatment of breast cancer.

Chronic inflammation is a key contributor to cancer development and progression [58]. Epidemiological studies have shown that chronic inflammation predisposes individuals to various types of cancer [59]. Cancer survivors with chronic inflammation may have an elevated risk of recurrence as a result of the effects of inflammatory processes on cell growth or the presence of cancer cells that induce inflammation [58]. Chronic inflammation is believed to contribute to the development and progression of breast cancer. Indeed, further observation of the C-T network shows that most of the high-degreed targets are associated with inflammatory mediators, such as COX-1 (PTGS1), COX-2 (PTGS2) and VEGF2 (KDR). There is a growing body of evidence that two primary cyclooxygenase (COX) isoforms (COX-1 and COX-2) play an important role in carcinogenesis and angiogenesis of human tumors [60]. COX inhibitors have potent activity in a model of highly aggressive breast cancer [61]. Epidemiologic and laboratory investigations suggest that non-steroidal anti-inflammatory drugs have chemopreventive effects against breast cancer due to their activity against COX-2, the rate-limiting enzyme of the prostaglandin cascade [62]. Epidemiological studies as well as early clinical trials also suggest that administration of either dual COX-1/COX-2 or selective COX-2 inhibitors may reduce the risk of cancer development [61]. Inhibition of COX-2 (and COX-1) results from the use of non-steroidal anti-inflammatory drugs and a case-controlled study has reported a reduction in the incidence of breast cancer in patients reporting a regular intake of these drugs [60]. VEGF2 is also a viable target for pharmacological intervention in cancer, and the aberrant expression of VEGF2 is a hallmark of malignant tumor development required for the colonization of endothelial cells that allow tumor nutrition [63]. Therefore, these high-degreed targets should be treated as the crucial ones of these herbs.

Furthermore, mu-type opioid receptor and delta-type opioid receptor are also targeted by candidate components from 20 herbs, which have the effect of sedation. Opioids, most common drugs used for treatment of cancer pain, are immunosuppressive, and therefore, they might directly and/or indirectly influence long-term cancer recurrence. For this reason, OPRM1 (mu-type opioid receptor) genotype may play a key role in both the short-term postmastectomy outcome and the long-term follow-up, becoming a new biomarker for breast cancer recurrence in patients suffering from chronic postmastectomy pain managed by opioid therapy [64]. In addition, OPRD1 (delta-type opioid receptor) is also reported to have sedative effect [65].

### T-P network and systems analysis

**Fig 5:**
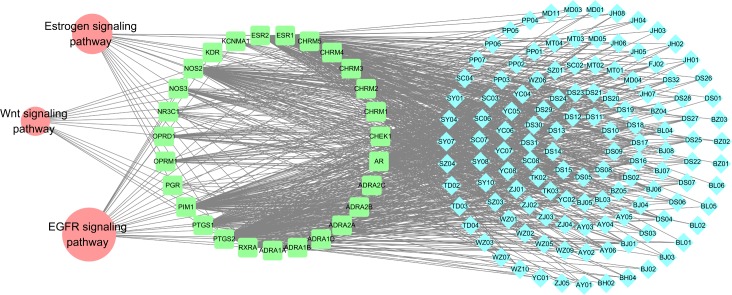
The T-P network. A link is created between a target and a pathway if the pathway is lighted at the target, where blue, green and red nodes represent compounds, targets and pathways, respectively. The information of pathways is obtained by mapping the target proteins to the KEGG pathway database.

Drug action is not only related to its targets, but also affects various metabolic enzymes, transporter proteins, as well as the downstream effects of drug action and pathways related to the specific disease. Most drugs act by binding to specific proteins, thereby changing their biochemical and/or biophysical activities, with multiple consequences on various functions. Multiple compounds can jointly perturb the same disease-related signal pathways [66]. For this reason, to understand the therapeutic mechanisms of a drug, it is also critical to identify the signal pathways its targets participate in. Thus presently, network-based analyses among compounds, targets and pathways are carried out to study the pharmacological mechanism of the herb in a systematical level as well as to unfold the main targeting proteins and signaling pathways in the process of treatment. As a matter of fact, for better elaborating the major pathways involved in herbal medicines for breast cancer therapy, we extract three canonical pathways that are highly associated with breast cancer from KEGG database (http://www.genome.jp/kegg/) to construct the target-pathway (T-P) network (Fig 5) with corresponding targets of herbal compounds.

The T-P network consists of 153 nodes and 865 edges, with blue, green and red nodes representing compounds, targets and pathways, respectively. As can be observed, most of the target proteins (27/33) appear in multiple pathways, indicating that these targets may intercede on the interactions and cross-talk between different pathways. Similarly, major pathways are also modulated through multiple target proteins, and many of them have been reported as suitable target pathways for breast cancer therapies, including the EGFR signaling pathway, Estrogen signaling pathway, and Wnt signaling pathway. The three pathways are interdependent with each other through the compounds, indicating that the herbal medicines may exert synergistic influences through multiple different pathways. Additionally, visually we find that most of compounds target more than one proteins involved in the same pathway or different pathways, which implies again the “multiple compounds, multiple targets” features of the TCMS.

Of all 33 candidate targets, 24 participate in the EGFR signaling pathway, indicating that the EGFR signaling pathway plays an important role in the treatment of breast cancer. Actually, aberrant epidermal growth factor receptor (EGF/EGFR) signaling is considered as a major characteristic of many human malignancies including breast cancer [67]. The signals mediated by EGFR can be deregulated by an increase in the amount of the receptor on the cell surface or by structural alterations of the protein that constitutively activates the signal pathway independent of its ligand [68]. The EGFR pathway is composed of several key transduction cascades, namely, those ones that are involved in the PI3K/Akt/mTOR, Ras/Raf/MAPK and JAK/Stat. For example, one of the pathways implicated in cell growth and metabolic regulation is the IGF1R/PI3K/Akt/mTOR pathway, which has been largely looked into the past decade as a target to treat breast cancer. The IGF1R activation by IGF1 leads to the phosphorylation of tyrosine residues in the intracellular domain of the receptor and phosphorylation of tyrosine and serine residues of the insulin receptor substrate (IRS) and Src which will activate the MAPK and PI3K/Akt/mTOR pathways. IGF pathway is regulated in many critical points, from ligand availability to negative feedback mechanisms exerted by mTOR [68]. As for its role in human cancer, preclinical models show that the IGF1R is frequently overexpressed by tumors, and mediates proliferation and apoptosis protection [69]. Thus, the EGFR pathway participates in cell metabolism regulation, in growth and survival, and changes in this pathway may contribute to various subtypes of breast cancer [68].

The second pathway, Estrogen, also plays an important role in the growth and differentiation of the normal mammary gland [70]. However, there is considerable evidence that estrogens are also mammary carcinogens in the breast, involving the metabolism of estrogen to genotoxic, mutagenic metabolites and the stimulation of tissue growth [50]. For instance, ERα, an estrogen-inducible transcription factor, is member of the nuclear receptor super family, the dysfunction of which accounts for 70% breast tumors [71]. Indeed, clinical and experimental studies have suggested an important role for Estrogen signaling pathway in the treatment of breast cancer and the reduction for estrogen deprivation of mammary tumors [70]. In breast cell, studies from several laboratories strongly support that estrogen also triggers signaling cascades typically linked to membrane receptors that possess tyrosine kinase activity or couple to heterotrimeric G proteins, such as MAPK, PI3K and PKB/Akt [72]. Therefore, given the role of this Estrogen signaling pathway in regulation of cell proliferation, mammary gland growth and development, estrogen is a major oncogenic factor and an attractive therapeutic target.

The third pathway, Wnt signaling pathway plays an important role in modulating the inflammatory response and the regulation of many cellular processes, including cell fate decisions and cell proliferation, and aberrant Wnt signaling also is associated with tumorigenesis [73, 74]. Presently, 12 compounds like WZ07 (Curcumenol), JH03 (Procurcumadiol), JH07 (Dihydrocurcumin), curcumin and its derivative, may be able to down regulate the Wnt signaling pathway through regulating β-catenin itself or downstream components, and then provide synergistic therapeutic effects to benefit patients [75, 76]. Many Wnt proteins act via a signaling pathway which always results in stabilization of β-catenin and consequent transcriptional activation of specific target genes. Mutations in β-catenin or other Wnt pathway components, which result in β-catenin accumulation, are found in a wide range of human cancers. Moreover, studies in animal model systems clearly demonstrate that activated Wnt signaling leads to mammary tumorigenesis [74]. Thus, Wnt signaling contributes to the genesis of cancers in a wide range of human tissues. These findings reveal the importance of Wnt signaling pathway in inducing breast cancer.

To sum it up, essential pathways for breast cancer mediate many of the characteristics of the malignant phenotype, such as increased cell proliferation, decreased apoptosis and metastasis. Since the three pathways are closely associated with epidermal growth factor receptor, estrogen receptor, and inflammatory, we speculate that the 20 herbs probably perturb the pathways, and thereby display anti-estrogen, anti-inflammatory, regulation of cell metabolism and proliferation activities. The three pathways are interdependent with each other through compounds, which further indicate that 20 herbs can exert synergistic influences on different pathways (Fig 5). In addition, a compound may target different proteins involved in the same pathway or different pathways, which also illustrates the mechanism of multiple targets for a TCM.

## Conclusion

Breast cancer is the most common malignancy in women. Traditional Chinese medicine has been practiced for three thousand years. This discipline is practiced worldwide that TCM may serve as a useful model for scientific inquiry since there is a standardized system of diagnostics and therapies. However, unlike conventional pharmacological medications used in western medicine, bioactive compounds and mechanisms of action of herbal medications have not been specified and measured precisely. Thus, in the present study, we have constructed a systems pharmacology approach including the OB screening, DL evaluation, target identification, and network pharmacology analysis, which combines the use of computational modeling and wide-scale text-mining methods, to elucidate the mechanisms of action of the most widely studied medicinal herbs for the treatment of breast cancer. The main findings are as follows:

1. Through OB and DL screening, 150 compounds out of 1618 components in 20 traditional Chinese medicines are identified as candidate compounds. 33 targets related to breast cancer are predicted to interact with the candidate compounds which generate the compound-target network. Based on the deep investigation of the function and the synergistic effect of each herb in the molecular/systems level, the combination principle of TCM formula can be explained as follows: *Radix Salviae* as monarch herbal medicine plays a principal role in therapeutic effect. 7 herbs are minister herbal medicine increasing the effect of *Radix Salviae*. The rest herbs serve as assistant and messenger drugs. All these results also explain why these herbs takes the ‘‘Jun-Chen-Zuo-Shi” as the rule of prescription.
2. Three pathways are closely associated with epidermal growth factor receptor, estrogen receptor, and inflammatory. Therefore, we speculate that the 20 herbs display anti-estrogen, anti-inflammatory, regulation of cell metabolism and proliferation activities by probably perturbing these pathways. The T-P network of these herbs constructed presently demonstrates that the herbal medicines may simultaneously target several pathways like EGFR, Estrogen and Wnt signaling pathways, thereby exhibiting synergistic benefits in breast cancer treatment.

## Supporting Information

S1 Table The Candidate Compounds and their Candidate Targets. (XLSX)

S2 Table The values of eight important molecular properties for DrugBank drugs and herbal compounds. (XLSX)

S3 Table GOBP enrichment analysis for 33 potential targets of active compounds. (XLSX)
